# Detection and quantification of offal content in ground beef meat using vibrational spectroscopic-based chemometric analysis

**DOI:** 10.1038/s41598-017-15389-3

**Published:** 2017-11-09

**Authors:** Yaxi Hu, Liang Zou, Xiaolin Huang, Xiaonan Lu

**Affiliations:** 1 Food, Nutrition and Health Program, Faculty of Land and Food Systems, The University of British Columbia, Vancouver, V6T 1Z4 BC Canada; 2 Department of Electrical and Computer Engineering, The University of British Columbia, Vancouver, V6T 1Z4 BC Canada

## Abstract

As less consumed animal by-product, beef and pork offal have chances to sneak into the authentic ground beef meat products, and thus a rapid and accurate detection and quantification technique is highly required. In this study, Fourier transformed-infrared (FT-IR) spectroscopy was investigated to develop an optimized protocol for analyzing ground beef meat potentially adulterated with six types of beef and pork offal. Various chemometric models for classification and quantification were constructed for the collected FT-IR spectra. Applying optimized chemometric models, FT-IR spectroscopy could differentiate authentic beef meat from adulterated samples with >99% accuracy, to identify the type of offal in the sample with >80% confidence, and to quantify five types of offal in an accurate manner (*R* ^2^ > 0.81). An optimized protocol was developed to authenticate ground beef meat as well as identify and quantify the offal adulterants using FT-IR spectroscopy coupled with chemometric models. This protocol offers a limit of detection <10% w/w of offal in ground beef meat and can be applied by governmental laboratories and food industry to rapidly monitor the integrity of ground beef meat products.

## Introduction

Ground or minced meat is an important meat product for food companies and consumers^1^ because of its wide usage in food products, such as sausages, burger patties, meatballs, meat fillings and numerous other dishes. However, mincing and grinding remove the morphological properties of meat, opening the door for adulteration. Although adulterations in animal-producing meat products have already existed for at least several decades or even hundreds of years^2,3^, concerns about the safety and integrity of these products has significantly increased since the horsemeat scandal emerged in Europe in January 2013. Similar cases have been noted worldwide, including Russia^4^, the United States^5^ and China^6^.

In brief, these incidents involve the substitution of high-priced or highly demanded meat products by cheaper and less-consumed meat of other animal species. Extensive studies have been conducted to identify this type of meat adulterations. Techniques including liquid chromatographic based methods, vibrational spectroscopies [*e.g*. Raman, infrared (IR) and nuclear magnetic resonance spectroscopies], and enzyme-linked immunosorbent assays all exhibited high capability to detecting adulterants from other animals^7–11^. Among all of these techniques, DNA barcoding is the most promising candidate with high efficiency and accuracy^12^. However, as DNA barcoding can only differentiate meat of different biological species, it cannot detect meat adulterated with cheaper meat cuts or the byproducts (*e.g*. offal and fat trimming) of the same species. According to the “Meat Hygiene Manual of Procedures” enacted by the Canadian Food Inspection Agency, any meat byproduct must be claimed on the label if it is mixed with the processed meat^13^. Because the consumption of animal offal is low among North American mainstream, an economically rewarding market outlet needs to be identified for extra economic benefit; and thus offal has high chance to be secretly included into ground meat by unscrupulous manufacturers. Therefore, it is critical to establish methods for the detection of offal in ground meat product.

As non-destructive and rapid analytical tools, mid-IR spectroscopy have been applied for the detection of beef meat mixed with beef offal by several research groups^9,14–16^. Various classification models were successfully applied in these studies for the identification of authentic beef meat and various types of pure offal^9,16^ or the differentiation between authentic beef meat from meat samples adulterated by offal^14,15,17^. However, no study has yet concluded the capability of spectroscopic techniques in identifying the specific type of offal in the adulterated beef meat samples. In some published works, partial least squares regression (PLSR) models were constructed to quantify the concentration of each type of offal individually in the adulterated meat samples with acceptable accuracy^9,16,17^. However, it was not feasible for these PLSR models to be applied to real samples because the specific type of adulterant in the adulterated beef meat sample was unknown and could not be identified using any classification model constructed in those studies. To accurately quantify the level of adulterant(s), the classification model(s) should be able to identify the specific type of adulterant(s) in a sample before predicting the concentration(s) using the corresponding regression model(s); alternatively, a single regression model could be trained to accurately quantify the concentration of the adulterant regardless of its identity. Therefore, to apply the vibrational spectroscopic-based chemometric analysis to samples in the marketplace, further modification and optimization of chemometric analysis and the combination of different chemometric models are required.

To the best of our knowledge, this was the first study to include both beef offal (*i.e*. beef liver, beef omasum, and beef honey comb tripe) and pork offal (*i.e*. pork heart, pork kidney and pork liver) as possible adulterants in ground beef meat and apply FT-IR for the analysis Moreover, this was also the first study of investigating the most appropriate sequence for the analysis of the identity and level of offal in adulterated beef meat. The established protocol to detect and quantify the adulterant(s) in ground beef meat is of great importance to meat industry and governmental laboratory to ensure the safety and integrity of ground beef meat products.

## Results and Discussion

### Exploratory analysis

**Figure 1:**
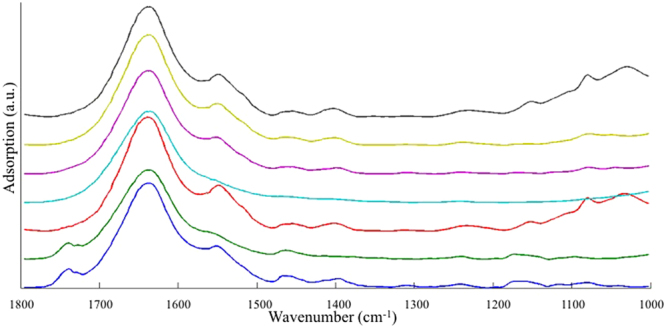
Averaged FT-IR spectra of each type of meat and offal samples. From the bottom to the top: beef meat, beef honey comb tripe, beef liver, beef omasum, pork heart, pork kidney and pork liver.

The averaged FT-IR spectra of different types of pure samples are stacked in Fig. 1 and the assignment of the peaks is summarized in Table 1. Differences in FT-IR spectra between offal and beef meat are in the region of 1765~1726 cm^−1^, 1572~1359 cm^−1^, and 1179~1000 cm^−1^, contributed by all macromolecules including lipids, proteins, nucleic acids and polysaccharides. Among six types of offal samples, beef liver and pork liver exhibited highly similar FT-IR spectral pattern; the spectral features between pork heart and pork kidney also could not be differentiated by simple visual inspection. In contrast, both beef omasum and beef honey comb tripe demonstrated some obviously different spectral patterns from the other four types of offal.

**Table 1:** Peak assignment of FT-IR spectra.

Peak assignment of FT-IR spectra			
wavenumber (cm^−1^)	assignment	wavenumber (cm^−1^)	assignment
1740	C=O stretching of lipid	1236	Phosphodiester stretching mode of nucleic acids
1726		1175	C-H bending of tyrosine
1638	Amide I group	1154	C-O stretching vibration
1548	Amide II group	1118	Symmetric stretching of P-O-C
1463	CH_2_ bending vibrations	1098	Symmetric stretching of PO_2_^−^ in nucleic acids
1399	CH_3_ symmetric bending	1080	
1310	Amide III protein secondary	1032	O-CH_3_ stretching of methoxy groups in polysaccharide

**Figure 2:**
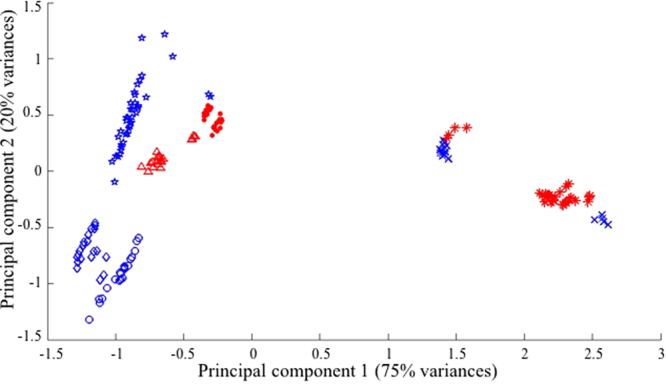
Score plots of principal component analysis of FT-IR original spectra. Each symbol represents one type of the sample: star, beef meat; diamond, beef honey comb tripe; cross-mark, beef liver; circle, beef omasum; triangular, pork heart; dot, pork kidney; and asteroid, pork liver. Symbols with blue colors represent tissue samples from beef, while red color symbols are samples from pork tissues.

Principal component analysis (PCA) is an effective tool to provide easy interpretation of the variances between data points in chemometric analysis^18^. Performing the linear combination of original variables (*i.e*. principal components), PCA transforms the axis (*i.e*. original variables), extracts the variances of the whole dataset, and concentrates the variances in the first several principal components^19^. Scores of the two or three newly formed principal components are then used to create 2-D or 3-D score plots to demonstrate the relationship between data points. PCA 2-D score plots of pure beef meat and pure offal samples were constructed using the original FT-IR spectra (Fig. 2). Data points of the same type of sample were tightly clustered and separated from others with a few outliers in the FT-IR spectroscopic-based PCA model. Mahalanobis distances were calculated to demonstrate the segregation of data points and the distances between the centroid of each cluster were higher than 9.8, given the common cut-off value of 3 to identify different clusters^20^.

PCA models using the 2^nd^ derivative transformed FT-IR spectra were also constructed (Fig. S1). However, the separation of different samples on the score plot of FT-IR spectra was not as good as that using the original FT-IR spectra. The separation of different samples on the score plots indicated the difference in spectra between various types of samples, and thus the datasets of the original FT-IR spectra demonstrated more obvious differences between each type of sample and higher similarity within each sample type. Therefore, the original FT-IR spectra dataset was used for further analysis.

### Classification analysis

Although spectra of pure offal samples could be separated from beef meat in the FT-IR spectroscopic-based PCA score plot, PCA could not separate adulterated samples with 10% offal from pure beef meat samples (data not shown). Therefore, analytical models with higher capability for class identification were investigated to differentiate authentic beef meat from adulterated beef meat. Briefly, supervised classification models using different algorithms including linear discriminant analysis (LDA)^21^, PCA-DA^22^, partial least squares (PLS)-DA^23^, K-nearest neighbors (KNN)^24^ and soft independent modeling of class analogy (SIMCA)^25^ were constructed and compared for FT-IR spectral datasets. Minor changes in the algorithm of each classification analysis were also investigated to optimize the performance.

#### Seven-class classification

Each dataset was first divided into seven classes, specifically beef meat and other six classes each containing samples of one type of offal (*i.e*. each class contains meat adulterated with one type of offal as well as pure offal). Wilks’ λ values were calculated for the original FT-IR spectral datasets to reduce the number of original variables by using Eq. 1. A total of 141 variables had Wilks’ λ values less than 0.5 and were selected for further analysis. Variables being selected were in the range of 1012~1109 cm^−1^, 1209~1252 cm^−1^, 1514~1560 cm^−1^, 1624~1664 cm^−1^ and 1724~1772 cm^−1^. These regions on the spectra were in accordance with the regions that demonstrated differences between offal and meat observed by visual inspection.

Seven-class classification models were constructed using those aforementioned algorithms and their derivatives for the calibration dataset of FT-IR spectra. A 10-fold cross-validation was performed for each classification method on the calibration dataset to avoid the overfitting of the model. In addition, prediction of new samples using each calibration model was performed to provide better assessment of the predictability of the calibration model. More than ten models were constructed for FT-IR spectral datasets. A good model should have low error rate as well as high accuracy, sensitivity, specificity and precision. Besides, models performing similarly in the calibration, 10-fold cross-validation and prediction have less chance to be overfitted and thus have higher potential to analyze new samples with accuracy^26^. When the performance was similar, the model offering the highest sensitivity and specificity for the beef meat group was selected because the clear separation between beef meat and adulterated beef meat is of the utmost importance that can protect the profit of both consumers and the producers. The best three classification models for FT-IR spectra were LDA, PCA-DA using the auto-scaled spectral dataset with 13 principal components, and PLS-DA using non-scaled spectra with 16 latent variables. Model parameters are summarized in Table S1. Being used for the prediction of new samples, all three models resulted in accuracy values greater than 80% and the averaged misclassification rate for each class was less than 20%. Among these three models, the LDA provided 100% specificity, sensitivity and precision for classifying authentic beef meat and thus recognized as the best model.

However, although the LDA model for FT-IR spectra provided 100% accuracy in identifying the authentic beef meat in the prediction dataset, there was a significant increase in the overall error rate in the prediction model than that of the calibration model (*i.e*. from 1% to 18%), indicating the existence of overfitting in this FT-IR LDA model. Moreover, the misclassification rate was high among different offal samples. Therefore, classification models separating samples into only three classes (*i.e*. authentic beef meat, samples with beef offal and samples with pork offal) were studied for a more accurate determination of offal adulteration.

#### Three-class classification

After dividing each dataset into three classes (*i.e*. authentic beef meat, samples with beef offal and samples with pork offal), Wilks’ λ values were calculated again for the calibration dataset of FT-IR spectra. Wilks’ λ values for FT-IR spectral calibration dataset were all above 0.5, and thus the selection criteria was increased to 0.75 with 65 variables in the wavenumber regions of 1168~1184 cm^−1^, 1454~1471 cm^−1^, 1545~1573 cm^−1^, and 1722~1778 cm^−1^ being selected. Variables that contributed to the inter-class variances were associated with proteins and nucleic acids.

The best three models for 3-class classification analysis of FT-IR spectra were LDA, PCA-DA using auto-scaled spectra with 14 principal components, and PLS-DA using mean-centered spectra with 14 latent variables. The parameters of the three classification models are summarized in Table S2. Based upon the parameters of beef meat class, the LDA model had the best performance (Table 2) with low overall error rate as well as 100% specificity and sensitivity for the beef meat class. The score plot can be found in Fig. S2. In addition, this model had extremely low error rate in identifying new samples and no significant changes occurred in the error rate and accuracy values in calibration, 10-fold cross-validation and prediction. Thus, this LDA model had a high potential for testing the market samples.

**Table 2:** The optimized models for the 3-class classification of FT-IR spectral data.

LDA model for FT-IR									
	calibration: error rate 1%, accuracy 99%			10-fold CV: error rate 3%, accuracy 97%			Prediction: error rate 4%, accuracy 94%		
class	specificity	sensitivity	precision	specificity	sensitivity	precision	specificity	sensitivity	precision
beef meat	100%	100%	100%	100%	100%	100%	100%	100%	100%
with beef offal	100%	97%	100%	98%	94%	98%	92%	96%	91%
with pork offal	98%	100%	97%	95%	98%	95%	97%	90%	96%
*#* of spectra in calibration dataset for beef meat, with beef offal and with pork offal are 40, 195 and 195, respectively.									
*#* of spectra in prediction dataset for beef meat, with beef offal and with pork offal are 15, 105 and 105, respectively.									

#### Identification of different types of beef and pork offal

After classifying samples into three classes, models that could identify either each type of beef offal or each type of pork offal were constructed. Based upon Wilks’ λ values < 0.5, 124 and 41 variables of beef offal FT-IR spectral datasets and pork offal FT-IR spectral datasets were selected respectively and used for further model construction. The selected wavenumber regions were 1002~1240 cm^−1^, 1700~1714 cm^−1^, and 1764~1795 cm^−1^ for beef offal and 1105~1182 cm^−1^ for pork offal.

SIMCA using mean-centered data with 2, 6 and 3 principal components for each class was the best model for the identification of the specific type of beef offal. LDA using non-scaled spectra had the best performance in recognizing the type of pork offal (Table 3). The score plot of this LDA model is shown in Fig. S3. Both models provided over 80% accuracy in predicting the identity of adulterant in new samples. SIMCA model for beef offal had 100% specificity and sensitivity in recognizing beef liver. All the misclassification occurred between beef honey comb tripe and beef omasum classes. Indeed, beef honey comb tripe and beef omasum are different parts of beef stomach and thus the chemical compositions could be similar. Therefore, SIMCA model was constructed again with beef honey comb tripe and beef omasum treated as one single class. The results of this 2-class beef offal SIMCA model using mean-centered spectra with 4 principal components are summarized in Table S3. All of the samples in the calibration dataset and prediction dataset were classified correctly. Thus, FT-IR spectroscopy could be applied to differentiate adulterated beef meat samples containing beef tripe from those containing beef liver when the adulteration level was above 10%. LDA model for pork offal had high accuracy in calibration and 10-fold cross-validation, but the accuracy significantly decreased in prediction, indicating the potential of model overfitting. The misclassification rate between pork heart and pork kidney classes was higher than that between any other combinations. Thus, a 2-class LDA model was constructed with pork heart and pork kidney treated as one single class. The error rate of the model in predicting new samples was decreased from 16% to 9% in this 2-class LDA model (Table S3).

**Table 3:** The optimized models for the 3-class classification of beef and pork offal using FT-IR spectra.

SIMCA model for beef offal using FT-IR spectra									
	calibration: error rate 15%, accuracy 82%			10-fold CV: error rate 17%, accuracy 83%			Prediction: error rate 20%, accuracy 80%		
class	specificity	sensitivity	precision	specificity	sensitivity	precision	specificity	sensitivity	precision
beef honey comb tripe	89%	75%	78%	88%	74%	75%	94%	51%	82%
beef liver	100%	100%	100%	100%	100%	100%	100%	100%	100%
beef omasum	88%	78%	76%	87%	75%	74%	76%	89%	65%
Each type has 65 spectra in calibration dataset and 35 spectra in prediction dataset									
**LDA model for pork offal using FT-IR spectra**									
	**calibration: error rate 2%, accuracy 98%**			**10-fold CV: error rate 5%, accuracy 95%**			**Prediction: error rate 16%, accuracy 84%**		
class	specificity	sensitivity	precision	specificity	sensitivity	precision	specificity	sensitivity	precision
pork heart	98%	98%	97%	98%	95%	95%	96%	71%	89%
pork kidney	99%	97%	98%	98%	94%	95%	84%	91%	74%
pork liver	100%	100%	100%	98%	97%	95%	96%	89%	91%
Each type has 65 spectra in calibration dataset and 35 spectra in prediction dataset									

### Quantification analysis

To provide straightforward quantification tool for adulterants in ground beef meat, FT-IR spectroscopic-based PLSR model was constructed to determine the level of adulterant regardless of its type. PLSR model with 10 latent variables extracted more than 95% of the total variances of FT-IR spectra, which was in accordance with the high reproducibility of FT-IR spectra. PLSR model for all adulterated samples with 11 latent variables was constructed and the performance of this model was further evaluated using 5-fold cross-validation and prediction dataset. *R* ^2^ and RMSE of calibration and 5-fold cross-validation was 0.96 and 0.07, respectively (Table 4), indicating high accuracy in quantifying the offal content in ground beef meat. However, *R* ^2^ and RMSE of prediction was 0.75 and 0.11, respectively, demonstrating some extent of overfitting of the PLSR model. Therefore, quantification of adulterant in ground beef meat without the predetermination of the type of adulterant could not provide accurate results.

**Table 4:** Partial least squares regression (PLSR) models for the quantification of offal using FT-IR spectroscopies.

	model	N^a^	n^b^	factors	RMSEC^c^	*R* ^2^	RMSECV^d^	*R* ^2^-CV	RMSEP^e^	*R* ^2^-P
FT-IR spectroscopy	quantification of offal	425	180	11	0.07	0.96	0.07	0.96	0.11	0.75
	quantification of beef offal	245	90	9	0.05	0.98	0.06	0.97	0.06	0.97
	quantification of pork offal	245	90	8	0.07	0.96	0.06	0.96	0.14	0.58
	quantification of beef honey comb tripe	75	30	6	0.03	0.99	0.04	0.98	0.05	0.96
	quantification of beef liver	75	30	6	0.03	0.99	0.03	0.99	0.03	0.95
	quantification of beef omasum	75	30	5	0.04	0.99	0.04	0.96	0.03	0.95
	quantification of pork heart	75	30	10	0.04	0.99	0.07	0.96	0.1	0.45
	quantification of pork kidney	75	30	9	0.03	0.99	0.04	0.98	0.07	0.94
	quantification of pork liver	75	30	6	0.03	0.99	0.04	0.99	0.07	0.81
	quantification of beef honey comb tripe & omasum	135	60	5	0.04	0.99	0.04	0.98	0.04	0.95
	quantification of pork heart & kidney	135	60	9	0.05	0.98	0.06	0.97	0.14	0.67

^a^N, number of spectra for calibration. ^b^n, number of spectra for prediction. ^c^RMSEC, root mean squares error of calibration. ^d^RMSECV, root mean squares error of cross-validation (9-fold). ^e^RMSEP, root mean squares error of prediction.

All the datasets for PLSR model were then divided into two parts, namely datasets containing beef offal and datasets containing pork offal. PLSR models were constructed separately for beef and pork offal using 9 latent variables and 8 latent variables, respectively (Table 4). Both models demonstrated high accuracy in calibration and 5-fold CV models (*R* ^2^ > 0.95 and RMSE < 0.07). However, only the PLSR model for beef offal had the capability in predicting the level of offal in new samples with high accuracy (high *R* ^2^-P and low RMSEP). Besides, the similar *R* ^2^ and RMSE values in the calibration, 5-fold cross-validation and prediction of the PLSR model indicated a very low potential of model overfitting. Therefore, PLSR model with 9 latent variables could be used to accurately quantify beef offal in ground beef meat.

According to the results of the classification models in section 3.2.3, the specific type of beef offal or pork offal could be identified with acceptable accuracy using the corresponding models. Therefore, PLSR models for each type of offal were constructed and the results are summarized in Table 4. PLSR models for beef honey comb tripe, beef liver, beef omasum and pork kidney all had *R* ^2^ > 0.94 and RMSE < 0.07 even in the prediction of new samples, and thus could all be used to provide accurate estimation of the concentration of specific type of offal in ground beef meat. The PLSR model for pork liver exhibited overfitting when only 6 latent variables were involved in the model, but *R* ^2^ and RMSE values of prediction were acceptable. However, the PLSR model constructed for pork heart demonstrated obvious overfitting, with *R* ^2^ decreased from 0.99 in calibration to 0.45 in prediction. Thus, it lacked the capability of predicting the concentration of pork heart in new unknown samples. In section 3.2.3, 2-class classification models that combined beef honey comb tripe with beef omasum and pork heart with pork kidney demonstrated higher prediction accuracy. Therefore, PLSR models combining the two types of beef offal and two types of pork offal were also constructed with the results shown in Table 4. The PLSR model for beef honey comb tripe and omasum had similar results to the PLSR models for these two types of offal individually, while the model for pork heart and kidney provided a higher accuracy in predicting the concentration of pork heart, but was worse than the one for pork kidney alone.

### Procedure for real sample analysis

For a new unknown sample, the level of the offal adulterant cannot be directly quantified because the PLSR model for all types of offal lacked the accuracy in prediction. Therefore, a classification model is required before performing the quantification analysis. Compared to the LDA model classifying samples into seven classes, the LDA model classifying samples into authentic beef meat, beef meat adulterated by beef offal, and beef meat adulterated by pork offal had higher prediction accuracy and thus could be applied first to test the authenticity of a new unknown sample.

If the sample was classified into beef offal class, a PLSR model for beef offal can be directly applied to quantify the concentration of the adulterant with high accuracy. Alternatively, SIMCA models could be used to classify the sample into three sub-classes with >80% accuracy or two sub-classes (*i.e*. beef honey comb tripe and beef liver treated as one class) with >99% accuracy. Then, PLSR models for each individual type of beef offal or the model for combined beef honey comb tripe and beef liver could be applied to quantify the level of adulterant. However, PLSR model for the three types of beef offal together had the highest *R* ^2^-P and lowest RMSEP. In the real analysis, determining the existence and concentration of the adulterants is usually more important than knowing more details about the type of adulterants. Thus, PLSR model for the quantification of all types of beef offal is recommended to use after the sample was confirmed to contain beef offal.

For samples that were classified into pork offal class, PLSR model for all types of pork offal could not be applied to determine the concentration because of its low predictability. However, the LDA model for pork offal provided adequate accuracy (*i.e*. >84%) in recognizing the specific type of pork offal. After acquiring the identity of the specific type of pork offal in the sample, corresponding PLSR model could be applied to quantify its concentration. However, if the sample was recognized as pork heart, the predicted concentration might not be fully accurate. Alternatively, LDA model classifying samples into two classes (*i.e*. pork heart and pork kidney combined) could provide higher accuracy (*i.e*. >92%) in determining the identity of the sample. Then, PLSR models for pork liver class as well as pork heart and kidney class could be applied accordingly. The accuracy in predicting the concentration of pork heart can be improved (*i.e. R* ^2^-P increased from 0.45 to 0.67) with the compensation of reducing the accuracy to quantify pork kidney (*i.e. R* ^2^-P decreased from 0.94 to 0.67). Taken all the factors into consideration, classifying samples into three classes followed by PLSR model for each type of pork offal is recommended.

**Figure 3:**
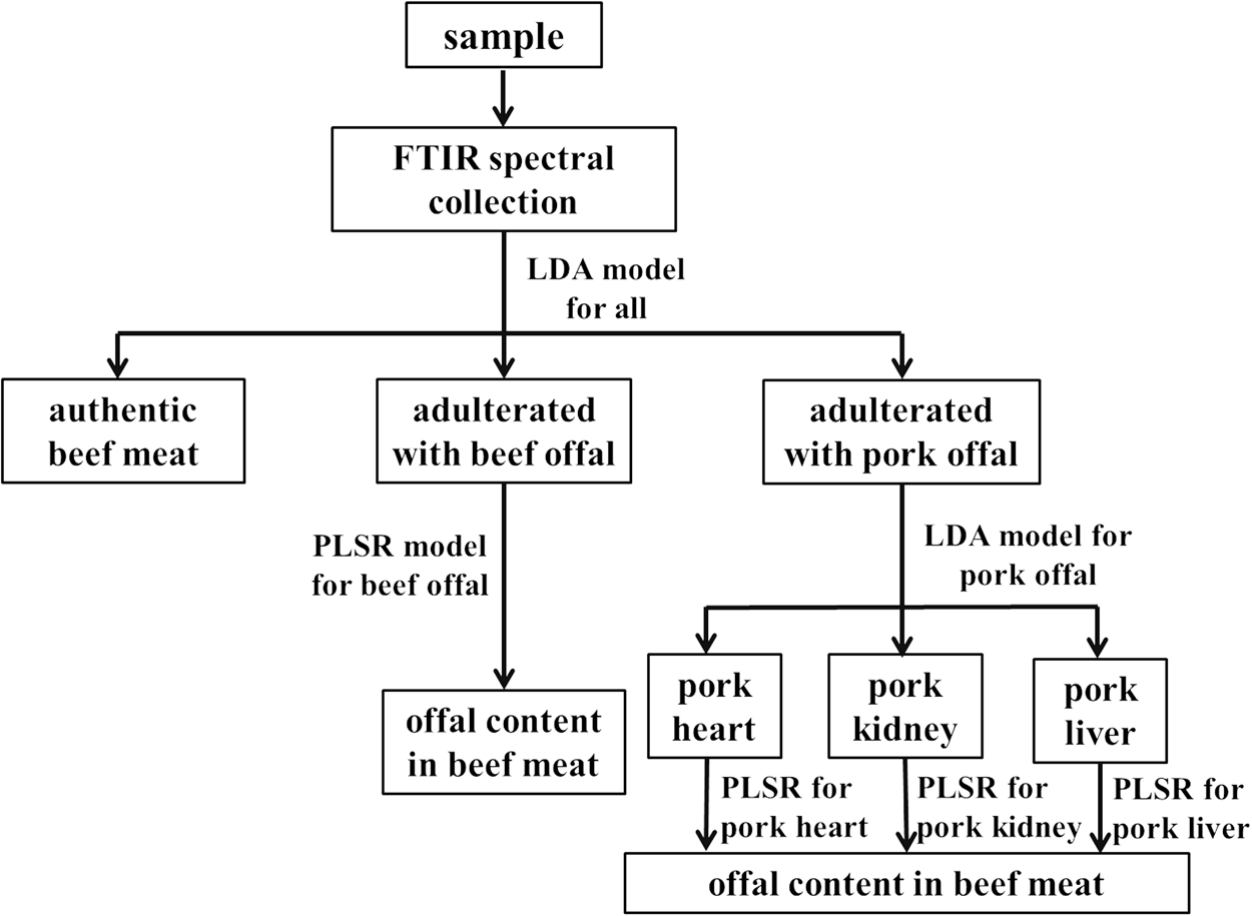
Illustration of the flow chart for the analysis of new ground beef meat samples.

The flow chart for 1) the differentiation of authentic beef meat from beef meat adulterated with offal and 2) quantification of the adulterant is shown in Fig. 3. By incorporating and arranging these spectroscopic-based chemometric models based upon the flow chart, a simple spectrum-to-answer interface can be designed using Matlab gui function to achieve an user-friendly application. The lowest concentration of offal in beef meat that can be differentiated from authentic beef meat using classification models was 10%. Therefore, the limit of detection (LOD) of offal in beef meat should be below 10% by using this protocol.

## Materials and Methods

### Preparation of meat and offal samples

Beef meat and offal samples were purchased from several local grocery stores in Metro Vancouver (BC, Canada). Samples included three types of beef meat cuts (*i.e*. beef ribeye, beef flank steak, and beef chuck steak), three types of pork offal (*i.e*. pork heart, pork liver, and pork kidney), and three types of beef offal (*i.e*. beef liver, beef omasum, and beef honey comb tripe). Each type of the sample had five biological replicates purchased on different days during September 2015 to September 2016 from different grocery stores.

After the purchase, samples were grounded individually using KitchenAid food processor with a meat grinder accessory, followed by storage at −20 °C until further processing or analysis.

### Preparation of adulterated samples

To significantly decrease the manufacture cost, large amount of beef meat replaced by offal is necessary. However, if the percentage of offal in the product is high, sensory attributes will change and can be easily identified by consumers. Therefore, samples contained beef meat replaced by offal at 10%, 25%, 33.3%, 50%, and 66.6% (w/w) were individually prepared. Three batches of ground beef meat and offal samples prepared as indicated in section 3.1 were selected randomly. The adulterated samples were prepared in triplicates by mixing ground beef meat samples with each type of offal sample thoroughly at five different concentrations. In total, 90 adulterated samples were prepared and tested directly or stored at −20 °C until further analysis.

### FT-IR spectral collection

Perkin Elmer (Waltham, MA, USA) model spectrum 100 FT-mid-IR spectrometer coupled with a Horizontal Attenuated Total Reflectance (HATR) sensor was used for mid-IR spectral collection. Briefly, beef meat, offal and adulterated samples were deposited directly onto the plate to fully cover the ZnSe crystal sensor without leaving any air bubble between the sample and the crystal top plate. Five FT-IR spectra for each individual replicate were collected at room temperature over the wavenumber region of 4000 to 550 cm^−1^ with a total of 3451 points and 16 accumulations. Spectra of pure samples and samples with various offal concentrations were collected randomly on different dates.

### Vibrational spectral preprocessing

OMNIC software version 7.0 (Thermo-Nicolet, Madison, WI, USA) was applied for baseline correction and Salvitzky-Golay 2^nd^ derivative of FT-IR spectra in the spectral fingerprinting region (*i.e*. 1800 cm^−1^–1000 cm^−1^). Representative beef meat FT-IR spectra before and after spectral preprocessing are shown in Fig. S4. Outliers were removed directly after the baseline correction. Spectra before the second derivatization was denoted as “original spectra” in the following content, while spectra after second derivatization were labeled as “2^nd^ derived spectra”.

### Chemometric analysis

Several chemometric analytical models were constructed to investigate the vibrational spectral features and the relationship between the spectral signals, type of adulterants and the concentration of adulterants. All the models were constructed using Matlab 2014a (MathWorks Inc., Natick, MA, USA) with either self-developed codes or the Classification Toolbox for MATLAB version 4.2^23^.

Containing hundreds or thousands of variables (*i.e*. wavenumber), vibrational spectra can be too complex for visual identification of the difference and similarity among samples. Therefore, PCA, an unsupervised analytical method that represents the most variances of the dataset with the fewest variables^27^ was applied to reveal the patterns in spectra of different samples.

Although PCA 2-dimensional or 3-dimensional score plots could demonstrate the clusters of different samples and thus is often applied for the purpose of categorization, the adoption of supervised classifiers in such studies is conceptually correct^28^. In the current study, classifiers including LDA, PCA-DA, PLS-DA, KNN and SIMCA were all tested individually on the FT-IR spectral datasets using none-scaled, mean-centered or auto-scaled spectral signals.

Achieving the same objective, the mechanism and principle of these classifiers to extract the information from the spectral datasets are different and thus suitable for different applications^29^. LDA is considered as the first multivariate classification technique and the most applied classifier^21^. By taking the linear combination of original variables, LDA transforms the high dimensional dataset into a few canonical variables (the number of canonical variables is the number of classes minus one) that maximize the ratio of inter-class variances to intra-class variances. Thus, the scores of the dataset projected on the new dimensions of canonical variates will be used to define the boundaries between classes. However, LDA can only be applied when the number of original variables is less than the number of the data points in the dataset. Therefore, variables selection is conducted by calculating the Wilks’ λ values for each variable using the following equation:

1 $$Wilks\mbox{'}\lambda =\frac{Intra-class\,variability}{Intra-class\,variability+intra-class\,variability}$$

Variables with λ value less than 0.5 indicate that the intra-class variances of these variables were smaller than the inter-class variances. Besides calculating the canonical variables, both PCA and PLS algorithms can be used for DA classifiers. Similar to LDA, both PCA-DA and PLS-DA define new variables by taking linear combination of the original variables (*i.e*. principal components of PCA and latent variables of PLS). However, in contrast to optimizing the ratio between inter-class variances and intra-class variances, PCA transforms the dataset into the directions maximizing the variances between each data points^22^. Instead of optimizing the variances of the dataset, PLS defines latent variables by treating the class identities as dummy variables and maximizing the covariance between spectral dataset and class identity^23^. KNN is another type of the traditional classifier^24^. First, multivariate distances (*e.g*. Euclidean distance and Mahalanobis distance) were calculated between each pair of data points. Then, the identity of each sample was defined by the k-closest data points around it. KNN has been commonly applied as a standard reference method for more advanced classifiers because it is based upon a non-linear algorithm, which is in accordance with those advanced classifiers. The last classifier applied in this study is a class modeling method, namely SIMCA. SIMCA contains multiple PCA models constructed for each class, respectively. Providing a new sample, the projections of this sample on each PCA model will be calculated. The multivariate distances of this sample to other samples in each PCA model will be used to define the identity^25^.

To provide the best predictability and avoid overfitting of the models^28^, all the samples were separated into two datasets, namely calibration dataset and prediction dataset. Specifically, the calibration dataset contains four replicates of each type of pure sample and three replicates of adulterated samples at 10%, 33.3% and 50% (w/w) levels (*i.e*. a total of 430 spectra); and the prediction dataset consists the remaining samples (*i.e*. a total of 225 spectra). In addition to training the model, calibration dataset was also applied to cross-validate the calibration model using a 10-fold cross-validation algorithm. Briefly, the calibration dataset was randomly divided into 10 groups and one group was treated as “unknown” to evaluate the predictability of the model constructed using the other nine groups^30^. This calculation was repeated for 10 times until every single group has been treated as the “unknown” sample. The average of the predictability of each repetition was calculated to better represent the performance of the calibration model. Parameters related to the performance of classification models were recorded, including the sensitivity (*i.e*. 1-type I error), specificity (*i.e*. 1-type II error), and precision for each class, as well as the error rate and accuracy of the overall model^31,32^. The equations for the calculation of each parameter are summarized here:

2 $$sensitivity=\frac{correctly\,classified\,sample\,in\,class\,C}{total\,number\,of\,sample\,belonging\,to\,class\,C}$$

3 $$specificity=\frac{correctly\,classified\,samples\,in\,classes\,other\,than\,C}{number\,of\,total\,samples\,belonging\,to\,other\,classes}$$

4 $$precision=\frac{correctly\,classified\,sample\,in\,class\,C}{number\,of\,all\,samples\,classified\,into\,class\,C}$$

5 $$error\,rate=1-\frac{sum\,of\,the\,sensitivity\,of\,each\,class}{number\,of\,classes}$$

6 $$accuracy=\frac{correctly\,classified\,samples\,in\,classes\,other\,than\,C}{number\,of\,all\,samples}$$

PLS regression (PLSR) analysis was also performed in the current study to provide tools for quantitative analysis of the concentration of offal in the adulterated beef meat. Being able to transform the two datasets to find their best fit, PLSR significantly reduces the number of variables in the spectral dataset and has been extensively applied in chemometrics to correlate the spectral signals with other feature(s), such as the concentration of an analyte in a complicated sample matrix^33^. Involving a supervised dimension-reduction method, PLSR often provides models with better quantification accuracy compared with other regression models, such as PCA regression^34^. Consistent to all supervised chemometric analyses, prediction dataset used to prevent model overfitting is inevitable. Again, all the samples were separated into two datasets as used in the classification models. *R* ^2^ and root mean squares error of calibration (RMSEC), cross-validation (RMSECV) and prediction (RMSEP) were individually calculated to evaluate the performance of the calibration models.

### Data Availability

The datasets generated during and analyzed during the current study are available from the corresponding author on reasonable request.

## Conclusion

FT-IR spectroscopy coupled with a variety of chemometric models were applied to detect and quantify offal adulterants in ground beef meat. With an HATR accessory, FT-IR spectroscopy was able to 1) differentiate authentic beef meat from beef meat adulterated with one of the six types of offal, 2) identify the specific type of offal adulterant, and 3) quantify five types of offal in an accurate manner. An optimized protocol for the analysis of ground beef meat using FT-IR spectroscopy was developed with a LOD lower than 10%. This protocol has a strong potential to be applied by governmental laboratory and food industry for the real world analysis. By developing user-friendly software including these spectroscopic-based chemometric models, analysis of the authenticity as well as the determination of the adulterant in ground beef meat can be completed in a few minutes.

## Electronic supplementary material

Supplementary information
