# Supplementary material for: Detection and quantification of offal content in ground beef meat using vibrational spectroscopic-based chemometric analysis
Source: Sci Rep. 2017 Nov 9;7:15162. doi: 10.1038/s41598-017-15389-3 (PMC5680338; doi:10.1038/s41598-017-15389-3)
Supplement: Supplementary file 1 — Supplementary information [file 41598_2017_15389_MOESM1_ESM.pdf]

Detection and quantification of offal content in ground beef meat using vibrational spectroscopic-based chemometric analysis

Yaxi Hu[1], Liang Zou[2], Xiaolin Huang[1], Xiaonan Lu[1*]

[1]Food, Nutrition and Health Program, Faculty of Land and Food Systems, The University of British Columbia, Vancouver, V6T 1Z4, BC, Canada

[2]Department of Electrical and Computer Engineering, The University of British Columbia, Vancouver, BC, Canada

Corresponding authors: Prof. Xiaonan Lu, xiaonan.lu@ubc.ca;

Table

Table S1. The best three models for the 7-class classification of all samples using FT-IR spectra

		LDA calibration			PCA-DA calibration			PLS-DA calibration		
class	# spectra	specificity	sensitivity	precision	specificity	sensitivity	precision	specificity	sensitivity	precision
meat	40	100%	100%	100%	100%	93%	100%	100%	97%	100%
beef honey comb	65	100%	97%	100%	96%	51%	70%	97%	68%	80%
beef liver	65	100%	100%	100%	100%	94%	100%	100%	100%	100%
beef omasum	65	99%	100%	97%	92%	72%	60%	96%	72%	75%
pork heart	65	99%	100%	97%	98%	97%	90%	96%	98%	83%
pork kidney	65	100%	97%	100%	93%	95%	72%	99%	92%	95%
pork liver	65	100%	100%	100%	100%	77%	98%	98%	95%	91%
		LDA CV			PCA-DA CV			PLS-CA 10 fold CV		
class	# spectra	specificity	sensitivity	precision	specificity	sensitivity	precision	specificity	sensitivity	precision
meat	40	100%	100%	100%	100%	93%	100%	100%	93%	100%
beef honey comb	65	97%	74%	81%	96%	48%	69%	97%	62%	78%
beef liver	65	100%	97%	100%	100%	94%	100%	100%	100%	100%
beef omasum	65	96%	83%	77%	91%	74%	59%	95%	69%	70%
pork heart	65	99%	96%	93%	98%	94%	90%	95%	98%	79%
pork kidney	65	99%	94%	92%	93%	95%	72%	99%	92%	92%
pork liver	65	99%	95%	96%	99%	75%	94%	98%	94%	91%
		LDA prediction			PCA-DA prediction			PLS-DA prediction		
class	# spectra	specificity	sensitivity	precision	specificity	sensitivity	precision	specificity	sensitivity	precision
meat	15	100%	100%	100%	100%	93%	100%	100%	60%	90%
beef honey comb	35	91%	77%	60%	92%	80%	64%	94%	57%	65%
beef liver	35	100%	100%	100%	100%	100%	100%	100%	100%	100%
beef omasum	35	95%	54%	66%	96%	66%	74%	92%	71%	63%
pork heart	35	98%	71%	89%	97%	83%	83%	96%	94%	82%
pork kidney	35	95%	86%	75%	96%	80%	80%	96%	86%	79%
pork liver	35	98%	86%	91%	99%	86%	97%	99%	86%	97%

Table S2. The best three models for the 3-class classification of all samples using FT-IR spectra

		LDA calibration			PCA-DA calibration			PLS-DA calibration		
class	# spectra	specificity	sensitivity	precision	specificity	sensitivity	precision	specificity	sensitivity	precision
beef meat	40	100%	100%	100%	100%	93%	100%	99%	100%	91%
beef offal	195	100%	97%	100%	97%	89%	96%	99%	96%	98%
pork offal	195	98%	100%	97%	91%	100%	90%	96%	99%	96%
		LDA 10-fold CV			PCA-DA 10-fold CV			PLS-DA 10-fold CV		
class	# spectra	specificity	sensitivity	precision	specificity	sensitivity	precision	specificity	sensitivity	precision
beef meat	40	100%	100%	100%	100%	93%	100%	100%	93%	100%
beef offal	195	98%	94%	98%	97%	88%	96%	98%	95%	98%
pork offal	195	95%	98%	95%	89%	96%	88%	96%	99%	95%
		LDA prediction			PCA-DA prediction			PLS-DA prediction		
class	# spectra	specificity	sensitivity	precision	specificity	sensitivity	precision	specificity	sensitivity	precision
beef meat	15	100%	100%	100%	100%	80%	100%	96%	93%	61%
beef offal	105	92%	96%	91%	95%	90%	94%	96%	91%	95%
pork offal	105	97%	90%	96%	91%	96%	90%	94%	90%	93%

Table S3. The optimized models for the 2-class classification of beef and pork offal using FT-IR spectra

SIMCA model for 2-class beef offal using FT-IR spectra									
calibration: error rate 0, accuracy 100%				10-fold CV: error rate 1%, accuracy 99%			Prediction: error rate 0, accuracy 100%		
class	specificity	sensitivity	precision	specificity	sensitivity	precision	specificity	sensitivity	precision
beef stomach	100%	100%	100%	98%	100%	99%	100%	100%	100%
beef liver	100%	100%	100%	100%	98%	100%	100%	100%	100%
LDA model for 2-class pork offal using FT-IR spectra									
calibration: error rate 0, accuracy 100%				10-fold CV: error rate 3%, accuracy 97%			Prediction: error rate 9%, accuracy 92%		
class	specificity	sensitivity	precision	specificity	sensitivity	precision	specificity	sensitivity	precision
pork heart & kidney	100%	100%	100%	97%	98%	98%	86%	96%	93%
pork liver	100%	100%	100%	98%	97%	95%	96%	86%	91%

# of spectra in beef stomach and pork heart & kidney group calibration and 10-fold CV is 130, while in prediction is 70. # of spectra in beef liver and pork liver calibration and 10-fold CV is 65, while in prediction is 35.

Figure

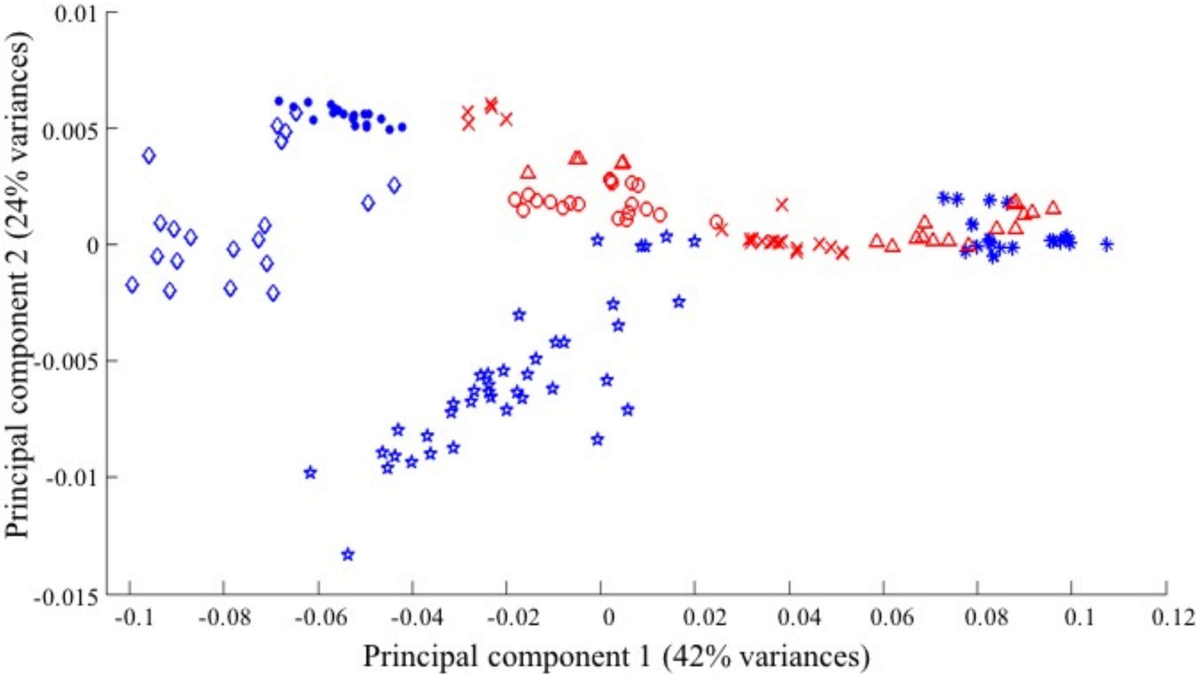

Fig. S1. Score plots of principal component analysis of 2nd derivative FT-IR spectra. Each symbol represents one type of the sample: star, beef meat; diamond, beef honey comb tripe; cross-mark, beef liver; circle, beef omasum; triangular, pork heart; dot, pork kidney; asteroid, pork liver.

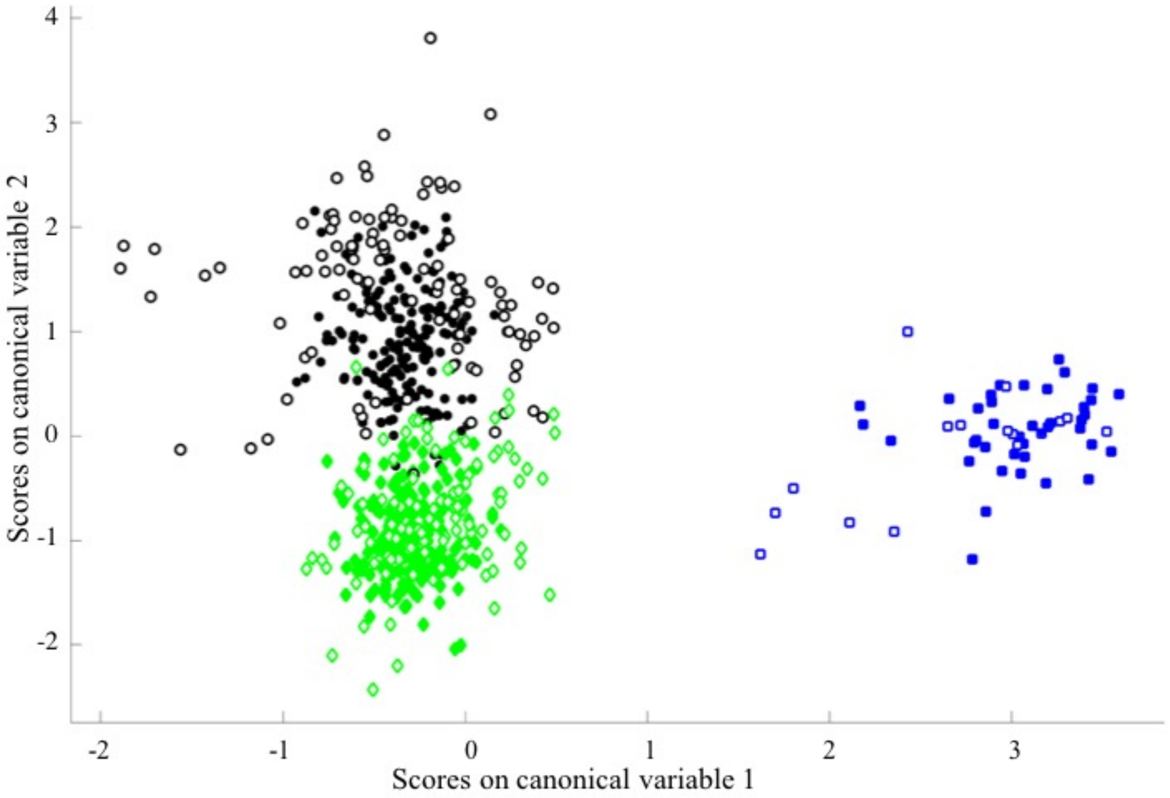

Fig. S2. Score plot of LDA model for 3-class classification of all samples. Solid blue squares, black circles, and green diamonds represent beef meat, beef meat with beef offal and beef meat with pork offal in calibration dataset, respectively. Hollow blue squares, black circles, and green diamonds represent beef meat, beef meat with beef offal and beef meat with pork offal in prediction dataset, respectively.

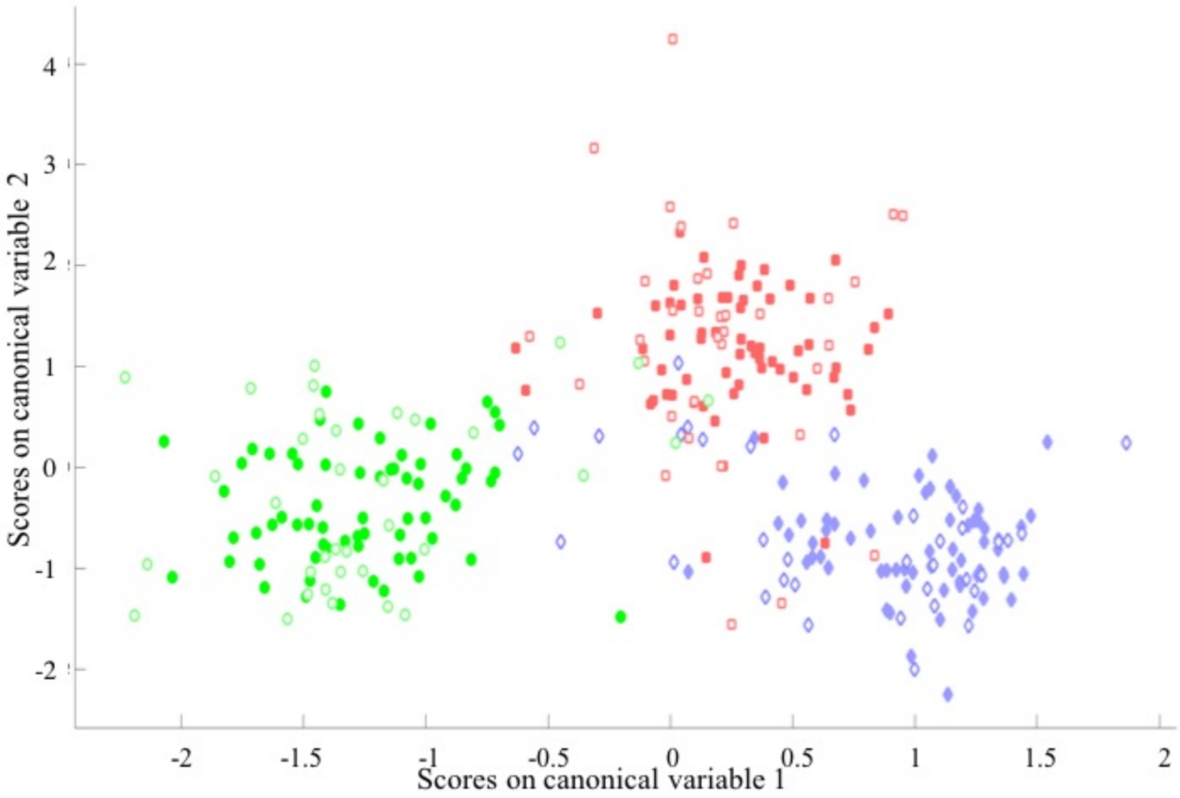

Fig. S3. Score plot of LDA model for 3-class classification of pork offal. Solid purple diamonds, red squares and green circles represent beef meat with pork heart, with pork kidney and with pork liver in calibration dataset. Hollow purple diamonds, red squares and green circles represent beef meat with pork heart, with pork kidney and with pork liver in prediction dataset.

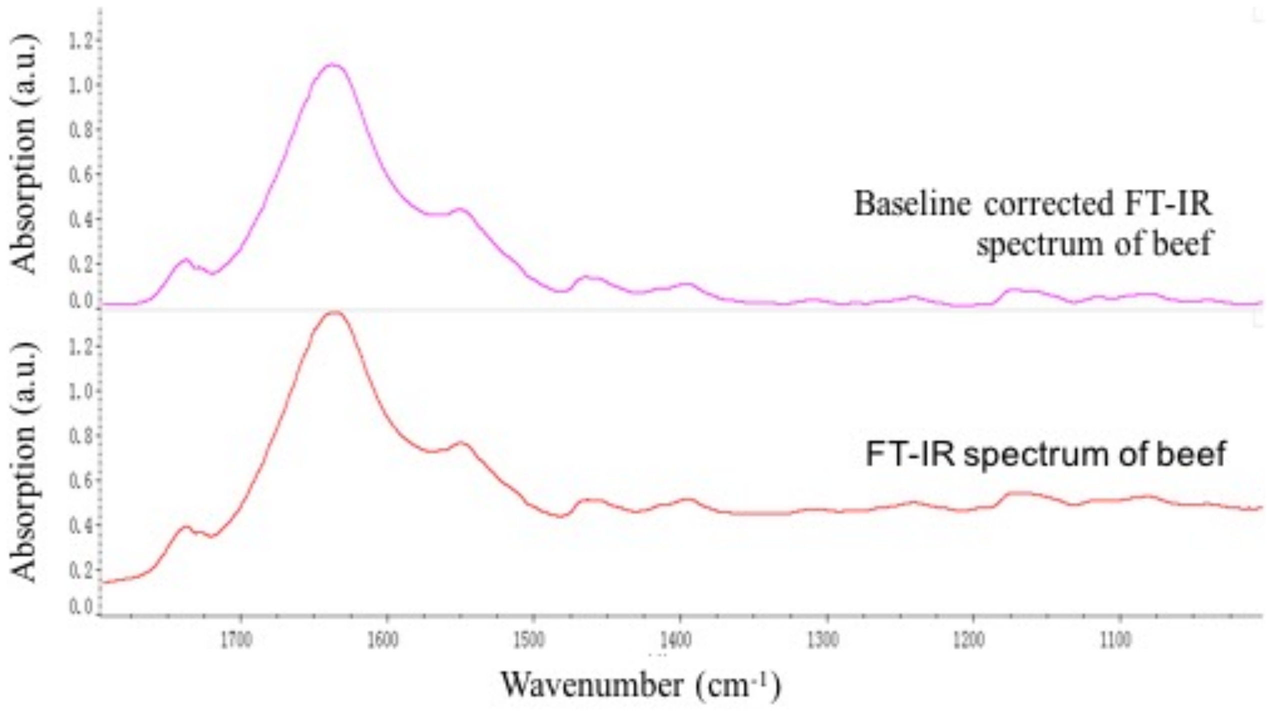

Fig. S4. Representative FT-IR (B) spectra of beef meat before and after spectral preprocessing.
